# Advanced Practice Nursing Education: Challenges and Strategies

**DOI:** 10.1155/2012/854918

**Published:** 2011-12-18

**Authors:** Cynthia Fitzgerald, Ira Kantrowitz-Gordon, Janet Katz, Anne Hirsch

**Affiliations:** ^1^College of Nursing, Washington State University, Spokane, WA 99210-1495, USA; ^2^Family and Child Nursing, University of Washington, Seattle, WA 98195, USA; ^3^College of Nursing, Seattle University, Seattle, WA 98122, USA

## Abstract

Nursing education programs may face significant difficulty as they struggle to prepare sufficient numbers of advanced practice registered nurses to fulfill the vision of helping to design an improved US healthcare system as described in the Institute of Medicine's “*Future of nursing*” report. This paper describes specific challenges and provides strategies to improve advanced practice nursing clinical education in order to ensure that a sufficient number of APRNs are available to work in educational, practice, and research settings. Best practices are identified through a review of classic and current nursing literature. Strategies include intensive interprofessional collaborations and radical curriculum revisions such as increased use of simulation and domestic and international service work. Nurse educators must work with all stakeholders to create effective and lasting change.

## 1. Introduction

National and international reports, including one published recently by the Institute of Medicine [1], describe the potential for advanced practice registered nurses (APRNs) to contribute to the provision of high-quality healthcare as part of comprehensive healthcare reform [2, 3]. Preparing APRNs for practice and fostering the role of APRNs in a variety of educational, clinical, and research settings are necessary steps toward achieving this vision. Given the current economic and political climate in the United States, however, success may be elusive. At present, a shrinking number of nurse educators carry an increasingly large responsibility for educating a declining number of APRNs [4, 5]. In many settings, outdated regulations, policies, and biases prevent APRNs from practicing to the fullest extent of their education, skills, and competencies [6–8]. Some US-based physician organizations have mounted campaigns aimed at discrediting APRN education and practice and decrying the potential of APRNs to provide cost-effective and clinically efficient care [9, 10]. 

While barriers to practice are significant, innovative approaches to clinical education and curricular transformation offer promise to nursing administrators, nursing educators, and practicing APRNs who are committed to preparing a highly qualified APRN workforce that will serve future generations of Americans. The rapid development and establishment of the practice doctorate has generated cautious enthusiasm among many nurse educators who are eager to help APRNs achieve their fullest potential in clinical practice. The purpose of this paper is to describe challenges in providing APRN clinical education and to propose achievable strategies for educating future APRNs to participate fully in transforming the United States healthcare system. We argue that the time is right to identify and implement educational practices that will lead to the optimal development of clinical skills, knowledge, and practice acumen and help meet the goals endorsed by national nursing organizations and set forth in the “*Future of nursing*” report published in 2011 [1]. While the IOM report is extraordinarily thorough, its scope does not include suggestions for specific strategies for improving APRN clinical education, a gap this paper seeks to fill.

## 2. Background

Advanced practice registered nurses include nurse practitioners (NPs), certified nurse-midwives (CNMs), certified registered nurse anesthetists (CRNAs), and clinical nurse specialists (CNSs). APRNs represent an underutilized source of quality health care providers [1]. Only 3.8% of the 2.4 million US registered nurses (RNs) are NPs, 0.3% are CNMs, 1.1% are CRNAs, and 0.9% (down from 1.2% in 2004) are CNSs [11]. While the nurse anesthetist was the first advanced practice role to emerge in the late 19th century, formal APRNs education programs did not start until the 20th century. The first nurse-midwifery program began in 1932 at the Maternity Care Association in New York, and in 1954, Rutgers University offered the first CNS graduate program with a specialty in psychiatric and mental health. The role of the nurse practitioner then developed in the 1960s with the increase in federal funding for advanced nursing education in order to fill the need for primary care providers [12]. Since the various roles have emerged, APRNs consistently provide high-quality, cost-effective patient care in a variety of healthcare settings [13]. Today, the majority of APRNs are employed in primary care settings, with most providing women's health, obstetrics, and mental health services [11]. One hallmark of APRN practice is the provision of care directed at illness prevention, health promotion, and improved patient care outcomes [14]. APRN practice represents one aspect of the nursing profession's ongoing efforts to provide high-quality healthcare to diverse populations. Overcoming barriers to APRN practice in today's healthcare environment will lead to improvements in health care for many, especially among traditionally underserved populations. 

We define many challenges associated with providing effective APRN clinical education, particularly in clinical practice settings. Our analysis of the challenges in Table 1 led us to identify innovative educational and programmatic strategies with potential to improve APRN education. The strategies we present include both internal (those related to educational institutions) and external (those related to social, political, and interprofessional practice issues) factors. 

## 3. Internal Challenges

For the purpose of this paper, we defined internal challenges as those existing within the profession and/or within educational organizations responsible for preparing APRNs for practice. When considering these internal challenges, we discovered, not surprisingly, that the literature was dominated by information about the critical role of the growing nursing and nursing faculty shortages. Clearly, not enough qualified nursing faculty are available to meet the nation's need for increased numbers of APRNs, and the projections describing future shortfalls are bleak [15, 16]. While the nursing faculty shortage has been well described in the literature, some aspects of it are germane in a discussion about APRN education, especially given the relatively large numbers of potential students unable to gain admission because of limited faculty resources [17]. 

Educational organizations find it increasingly difficult to attract qualified APRNs willing to serve in faculty roles. The demand for APRNs in both educational institutions and in a variety of practice settings has increased simultaneously, but educational institutions are disadvantaged by their inability to offer competitive compensation packages. Constrained budgets result in compressed salaries throughout higher education systems, increasing the gap between salaries available in practice and those offered for teaching positions. 

When APRNs do pursue education at the PhD level, they often graduate only to face the reality of the tenure process in research-driven educational institutions. Emphasis on the role of faculty in conducting research and generating research-related revenue limits the availability of PhD-prepared APRN faculty to participate in direct clinical supervision of APRN students. One result is that the primary responsibility for APRN clinical education falls to faculty not eligible for tenure [18] and whose salaries are typically lower than those available for APRNs in clinical practice [19]. Educational institutions without established faculty practice plans face additional barriers for supporting and retaining faculty who need to practice to maintain certification and licensure, in addition to teaching and meeting tenure criteria. 

As many schools of nursing transition to the Doctorate of Nursing Practice (DNP), existing advanced practitioner faculty without a doctorate may find that they are underqualified [20]. Institutional requirements for supervisory committees of doctoral students may require faculty to hold equivalent doctorates, and supervision of DNP students may increase faculty workloads. PhD-prepared nursing faculty may lack the advanced practice qualifications to teach specialty content in APRN programs. Smaller educational institutions may not have the institutional structures or additional faculty necessary to support the development of DNP programs [21]. While the development of DNP preparation and practice offers much promise for preparing the future workforce, the transition process may temporarily exacerbate the shortage of available clinical faculty and result in decreased numbers of APRN graduates. It is too soon to tell whether these transitional challenges will affect the quality of APRN clinical education. The net result may be additional reductions in the available supply of APRNs at precisely the time when they are most needed to address the challenges of healthcare reform in the US [21].

The number of annual graduates from APRN programs has fallen from a peak in 1998 [17]. This decline is multifaceted, relating to a variety of barriers facing nurses who might otherwise pursue graduate education. Admission to APRN educational programs can be difficult. As many as 17% of graduate nursing programs are highly selective, and there are insufficient openings for qualified applicants [22]. Program costs present challenges to potential applicants whose educational plans are altered by the recent economic downturn in the US as well as by declines in available employer tuition-reimbursement programs; in 2009, 15% of masters of nursing programs cited affordability as a commonly stated reason for students not enrolling [22]. Program location can be a deterrent to nurses who are place bound by responsibilities to support family and provide income. Although the need for more APRNs in rural communities is critical, APRN programs are less accessible to nurses in rural areas, where there are fewer nurses, and nurses must contend with lower salaries and longer commutes [23]. In some areas, there are vacancies in some nursing programs, while others may turn away qualified applicants. Additionally, there are significant shortages of Hispanic, Native American, and men in nursing and in APRN programs. White, non-Hispanic women make up over 83% of APRN nurses [11]. The result is a professional nursing community that does not reflect the diversity of the US population [24]. 

Since World War II, educational programs offering Associate Degrees have proliferated and graduates of those programs have become Registered Nurses (ADNs) in increasing numbers. In turn, this internal challenge has influenced the shortage of APRNs, given that nurses prepared in ADN programs are less likely than bachelor's prepared nurses to obtain graduate degrees [4]. If ADNs do pursue graduate education, time to completion of an APRN program expands, given the requirement for ADNs to complete bachelor's education before entering a graduate nursing program. Such problems clearly bring the APRN supply needs back to nurse educators and leaders at all levels.

## 4. External Challenges

The primary challenge facing APRN education from outside educational institutions is the limited number of available clinical sites and preceptors [22]. To increase the number of APRNs prepared to practice independently and to the fullest extent of their scope of practice, nursing education programs must increase both the number and quality of available preceptors and sites. Since many existing faculty practice settings are inadequate to meet this need, educational institutions must rely on cooperative, volunteer community preceptors. There is a shortage of APRN preceptors, particularly in acute care or hospital-based specialties (i.e., CNMs, neonatal nurse practitioners (NNPs), and acute care nurse practitioners). Often, APRN specialties require that preceptors hold the same specialty certification. For example, certified nurse-midwives (CNMs) must provide education to CNM students [25]. While there is a great need for APRN graduates to serve rural areas, there are even fewer preceptors and role models available in these underserved locations.

The limited supply of potential preceptors and clinical sites is exacerbated by competitive forces. Medical resident preparation dominates the use of available clinical sites in hospitals. Federal funding through the Medicare program supports resident education, but not APRN preparation. In many academic medical centers, APRNs are employed for medical student and resident education, further reducing the field of potential preceptors for APRN students [26]. Nursing educational institutions are concentrated in large urban areas near hospitals and may compete with other nursing educational institutions for clinical sites and preceptors.

State regulations and specialty certification agencies place additional requirements on educational institutions that further limit the capacity to prepare APRN students. Direct supervision of students limits the number of students individual preceptors may have at any given time. The requirement for low student-faculty ratios in clinical courses makes APRN education expensive. For example, the National Task Force on Quality Nurse Practitioner Education recommends faculty-to-student ratios of 1 : 6 in situations where there is indirect clinical supervision [27]. Requirements for supervised student clinical practice in most APRN programs are typically established at a minimum of 500 hours, and the DNP requires at least 1000 hours of clinical practice [19]. This increase in DNP student practice hours will increase the need for qualified and willing preceptors.

The limited availability of national funding poses a significant external challenge to successful APRN education. Increasing the capacity of educational institutions to educate APRNs requires additional funding. The current prioritization for medical education and residency training through federal support makes increasing funding for nursing education difficult. Furthermore, current research funding priorities by the National Institute of Nursing Research do not support the investigation of nursing education issues, nor do they support research about the implementation of innovative practice education models at the graduate level. In many research organizations, nursing faculty pursuing academic careers and tenure are discouraged from pursuing clinical education research as a funded line of inquiry. Among potential APRN preceptors, there may be a lack of willingness to precept APRN students due to a lack of incentives beyond the ideals of serving the profession. Most educational institutions are unable to compensate preceptors financially for their teaching roles and are limited in the nonfinancial benefits they may provide preceptors such as faculty titles and access to educational resources. Potential preceptors may see the challenges to practitioner productivity or the additional time commitments of being a preceptor as disincentives to assuming the role. The lack of formal preparation and support for the teaching role may further discourage APRNs from being a preceptor. While direct or graduate entry training is increasingly used as a mechanism for increasing the supply of APRN graduates, potential preceptors may be resistant to training students with little or no health care experience.

The final challenge to increasing the preparation of APRNs is closely tied to the profession's relationship with the citizens who are served. Nursing continues to be a profession dominated by Caucasian women, a limitation that affects the profession's negotiation of relationships with other more male-dominated professions. In addition to the chronic underrepresentation of men, diverse populations, and rural inhabitants in the nursing workforce, advanced practice nursing continues to contend with an identity crisis among the US population as a whole, who suffer from a knowledge deficit regarding the skills and abilities of APRNs. Historically, nurses work at the direction of physicians, and cultural and occupational patterns that reinforce this dependent relationship are slow to change. While it is not clear the American Medical Association's efforts to counter the IOM's Future of Nursing Report will be entirely successful [28], the lack of support for full-scope APRN practice from this influential organization is disappointing to those with a vision for the provision of collaborative care in an efficient and effective interprofessional model. Negotiating a new position in health care for nurses and APRNs will continue to be complicated by gender politics as well as power positioning.

## 5. Strategies and Solutions

The IOM report presents an unparalleled challenge to nursing educators, that is, to foster the development of an “improved education system that promotes seamless academic progression” [1, page 164]. Significant innovation and change are needed to accomplish this vision and to increase the number of APRN graduates. While some of what is required must be implemented on a nation-wide scale, there is strong potential for nursing education programs to implement local and regional strategies that will increase the numbers of APRN graduates prepared to practice at the fullest extent of their education and licensure. 

In preparing this discussion of strategies and solutions described in Table 2, we considered our own experience as educators in graduate nursing programs and explored recommendations from multiple authors describing approaches that have been successful in enhancing the education of APRNs. Taken individually, each of these strategies has the potential to help programs make incremental improvements in the recruitment, retention, and preparation of graduate nursing students. In combination, these strategies offer the promise of helping nursing education affect transformation in the preparation and practice of APRNs. 

For the purposes of this paper, internal strategies are those that can be undertaken within nursing education programs and the universities that house them, while external are those that reflect some level of engagement with other organizations including other nursing education programs and healthcare organizations. 

### 5.1. Internal Strategies

As noted above and in the IOM report, the expansion of advanced nursing education programs is hampered by a faculty shortage that represents the convergence of multiple factors. These include supply-side problems related to the nursing shortage itself as well as to competitive factors that reflect, among other things, the relatively high cost of graduate nursing education when compared to the earning potential of nurse educators. Like prelicensure nursing education, advanced practice nursing education is resource intensive, requiring sophisticated laboratory settings, computer equipment, and high faculty-to-student ratios. 

One approach with potential to aid in the nursing faculty shortage and to make more clinical resources available for APRN education involves internal efforts by educational institutions to develop and strengthen collaborative partnerships. The American Association of Colleges of Nursing [16] and the Robert Wood Johnson Foundation [29] recommend that educational organizations work with one another as well as with hospitals and healthcare organizations to develop innovative capacity expanding approaches for preparing nurses and nurse educators and to foster the expansion of nursing education programs. These programs are likely to be costly, but if the benefits can be well-described, educational institutions, hospitals, and healthcare organizations may be willing to invest in their success. As one example of innovative collaboration between university programs, Siewert and her colleagues from the University of Iowa College of Nursing report on collaborative efforts with the University of Missouri at Kansas City that allows for dual enrollment of neonatal nurse practitioner students and helps to optimize faculty resources and enhance student learning opportunities at both institutions [30]. An innovative array of academic and service partnerships linking Bassett Medical Center in Cooperstown, New York, with educational programs at the State University of New York Institute for Technology in Utica, New York now offers tuition support for advanced practice nursing preparation with an emphasis on improving care in a large rural community [31]. These programs and others like them offer much promise in addressing faculty shortages and other challenges while offering innovative contemporary APRN education to place-bound students.

In almost every aspect, curriculum, teaching, and learning must undergo radical transformation, as Benner and her colleagues asserted in 2010 [32]. Nursing programs have traditionally been content driven, but the needs of students and faculty are changing along with those of the workplace [1]. At the core of these new and revised curricula is an emphasis on integrating established educational and professional competencies with educational strategies that encourage problem solving and that enhance students' critical thinking abilities. Such curricula will encourage the simultaneous development of innovative learning activities, ensure effective student evaluations, and provide clinical experiences that emphasize the optimization of student practice outcomes [33]. Competency-based education may have additional advantages including the development of more learner competence, confidence, and compassion [34, 35].

Problem-based learning can be integrated within a competency-based framework or as a stand-alone strategy to enhance the development of critical thinking and hypothesis-testing skills [36, 37]. Problem-based learning (also known by other terms with slightly different applications, including case-, practice-, or concept-based learning) helps students ground learning in relevant clinical experiences [38, 39]. As students engage closely with faculty in exploring new concepts and identifying new solutions, the process of discovery can lead to the development of improved clinical judgment [40]. 

The use of simulation in nursing education is becoming increasingly popular for its ability to enhance the critical thinking of advanced practice nursing students and because it provides a useful evaluative tool for faculty [41]. Through the use of high-fidelity computerized simulation models, APRN students safely develop new knowledge and skills about high-risk, low-volume practices [42]. Other simulation activities involving scripted patients or rotation through skill-based practice stations in laboratory settings also offer enhanced opportunity for student learning and faculty participation. Clinical simulation activities can add greater value by linking APRN students with medicine, pharmacy, and rehabilitation students across the health sciences [43]. 

Interprofessional education offers the potential to enhance efficiency in the provision of clinical education for all students [44] and fosters collaborative practice beyond the educational period. Success has been demonstrated when APRN education has been integrated with specialty and generalist physician practice in a mental health practice setting, as described by Roberts and her colleagues [45] and likely has much potential to improve education and patient care in a variety of other settings. While mistrust by physicians of the APRN role threatens to constrain the development of collaborative educational models, the promise of interprofessional education also has the potential to unite APRN and physician practice. Such efforts to integrate education and training hold much promise for the US healthcare system as a whole. 

Distance education helps create opportunities for otherwise place-bound nurses to pursue graduate studies to become APRNs by extending the reach of nursing education programs beyond traditional boundaries. Improvements in online course management software and evidence-based distance teaching pedagogical approaches provide a foundation for the asynchronous delivery of high-quality and engaging course content. The use of streaming media and a wide range of unified communication technologies (e.g., video cameras, instant messaging, web-connected whiteboards, etc.) enhance faculty-student and student-student engagement. Despite the obvious challenges of providing adequate supervision for APRN students who may be completing coursework from remote areas and with little direct faculty contact, the rewards of accessing optimal professional education using distance education technologies can be great for place-bound students living in underserved communities. To help these programs and students to succeed, educational programs can develop innovative faculty hiring agreements, hiring APRNs who live in the students' home communities to provide supervision for didactic learning experiences as well as for clinical practice and evaluation. The education and support these faculty members may require can be provided in part by professional development or continuing education programming.

### 5.2. External Strategies

Not all responsibility for enhancing advanced practice nursing lies with classroom or faculty-driven learning activities. As the number of available clinical sites and preceptors has declined, the need to consider effective alternatives for APRN clinical education has increased. Nursing education programs must “aggressively pursue alternative clinical learning sites and experiences” if they want to assure that students participate in appropriate patient-centered learning activities [46].

The development of partnerships with a broad range of community organizations and providers can create mutual benefits and provide additional learning opportunities for APRN students. While faculty may believe that an ideal clinical placement would pair students with preceptors in one-to-one relationships with clients arriving at set appointment times, there may be great value in developing partnerships with agencies and individuals who provide care in different models and settings [47]. The development of community partnerships with a service-learning framework can provide APRN students with innovative opportunities to engage in health promotion, physical and mental health assessments, and intervention with individuals who might not otherwise receive healthcare services in a given setting. For example, assignment of students to a correctional facility could offer students the opportunity to engage with individuals in need of health assessment or behavioral intervention [48], even in the absence of a formally organized on-site health clinic. Assigning students to work with clients through a variety of community agencies can enhance learning opportunities for APRN students and improve care for individuals seeking nonhealthcare services such as meal delivery or day care [49]. Facilitating student engagement in homeless centers can provide a variety of learning opportunities while serving to increase student understanding of social conditions and mental illness [46]. These innovative learning opportunities can provide students with opportunities to build personally meaningful collateral skills even when the emphasis is on accomplishing practice-related learning objectives [50, 51]. 

In 2004, Connolly and her colleagues described the innovative creation of a collaborative approach to nursing education [52]. Although writing about associate degree nursing education, key concepts have the potential for application in advanced practice education. These include the introduction of interprofessional collaboration that links nursing, medicine, and allied health personnel education within single community health settings, allowing the development of knowledge and skills that are essential to advanced practice nursing. 

Academic health centers that integrate faculty practice opportunities with clinical education experience opportunities may well provide ideal environments for APRN education. Not all graduate nursing programs are situated on campuses that house such centers, however. Heller and Goldwater suggest that the development of innovative patient-driven programs, designed to improve access, may also offer enhanced clinical education opportunities for advanced practice students [53]. Their experience with the development of a mobile clinic offering primary care services by APRNs and their supervising faculty, dubbed the “Wellmobile,” illustrates a comprehensive and innovative approach to clinical care. In addition to providing a structured environment that places emphasis on the clinical education of APRN students, the “Wellmobile” also offered students the opportunity to develop strong business and management skills [53]. 

Although they can be costly and somewhat difficult to coordinate and offer, domestic and international healthcare missions do offer APRN students and faculty innovative opportunities to provide care to the underserved. While many available international opportunities are useful for student enrichment alone, with secure funding, careful planning, and rigorous attention to the management of learning and evaluation, successful programs can extend clinical education beyond local limits [3]. Participation in mission-driven clinical experiences offers students opportunities to provide care for vulnerable populations and can serve as cultural immersion experiences, enriching students' cultural competence. They may also provide opportunities for students to develop skills in leadership and practice inquiry, cornerstones of DNP practice. 

Finally, funding must be made available to support the vision that advanced practice nurses will assume a large measure of responsibility for the success of healthcare reform in the United States. Improvement in the healthcare system requires the collaborative effort of many disciplines. At present, the current “system of medical education and graduate training… is not aligned with the delivery system reforms essential for increasing the value of health care in the United States.” [54, page 103] The current system of funding graduate medical education does not provide sufficient resources to support the education of nurses in clinical practice settings. While it is typical for medical residents to be supported with salaries, stipends, living allowances, and even resources such as equipment and textbooks, responsibility for APRN clinical education rests solely with the students themselves. Educating an effective nursing workforce is a responsibility that must be shared by nursing programs, academic institutions, and government agencies with support from policy makers who will stand firm in sponsoring a coherent and appropriate approach to the education of a collaborative workforce [55]. It will not be sufficient to simply provide increases in available loans or to improve loan repayment programs; for APRN clinical education to be on par with medical education, nursing classroom and clinical education must receive full financial support. Further, there must be improvements in Medicare compensation for services provided by APRNs, including those related to performance as clinical preceptors and research mentors. Funding for improved and financially supported residency programs for APRNs could come from federal programs that accept a mandate to provide healthcare services to all citizens or that compensate physicians at greater rates than APRNs for the provision of equal services [56].

## 6. Conclusions

The Institute of Medicine Report on The Future of Nursing [1] calls for increasing the supply of highly educated and clinically skilled APRNs who can practice to the fullest possible extent of their scope of practice. Clearly, APRNs have the potential to contribute to the provision of high-quality healthcare as part of comprehensive healthcare reform in the United States. If this vision is to be accomplished, however, numerous challenges inherent in the current APRN educational process and barriers in the practice environment must be overcome. This paper has identified challenges that specifically hinder the clinical education of APRNs and proposed strategies and solutions to help educational institutions address them. In preparing this paper, we considered our personal experience and explored the literature describing innovative approaches and strategies that have been successful for others. These approaches to APRN clinical education can affect a radical transformation in the preparation of APRNs and help ensure the healthcare needs of US citizens are met by a diverse and collaborative workforce of professionals united in a vision to optimize the practice potential of all practitioners. It is imperative that nurse educators work with all stakeholders to improve the education of APRNs through the identification and implementation of best practice clinical education strategies designed to overcome the current barriers to the provision of high-quality clinical experiences.

## Figures and Tables

**Table 1:** Challenges to effective APRN clinical education.

Internal challenges	
High enrollments	
Increasing demand for nurse educators	
Noncompetitive educational salaries	
Difficulties associated with tenure-track research positions	
Proliferation of DNP programs	
Increasing faculty workloads	
Declining graduation from APRN programs	
Difficulties accessing education in rural communities	
Lack of APRN workforce diversity	

External challenges	

Limited number of clinical sites and preceptors	
Competitive forces (APRNs teaching residents)	
Concentration of educational programs in urban areas	
Regulatory agency and specialty certification requirements	
Limited national funding for clinical education research and preceptor compensation	
Demographic mismatch between nursing and populations served	
Gender politics and power relationships	

**Table 2:** Solutions and strategies.

Collaborative partnerships between educational institutions	
Collaborative partnerships between educational institutions and healthcare organizations	
Radical transformation of curricula to support competency-based and problem-based learning	
Increased use of simulation	
Interprofessional education	
Distance education	
Community partnerships with social service agencies	
Innovative patient-driven programs	
Domestic and international mission work	
Federal funding for APRN clinical education on par with graduate medical education	
